# Impact of the Dropping Function on Clustering of Packet Losses

**DOI:** 10.3390/s22207878

**Published:** 2022-10-17

**Authors:** Andrzej Chydzinski

**Affiliations:** Department of Computer Networks and Systems, Silesian University of Technology, Akademicka 16, 44-100 Gliwice, Poland; andrzej.chydzinski@polsl.pl

**Keywords:** TCP/IP networks, bufferbloat, dropping function, packet losses, burst ratio, queueing model

## Abstract

The dropping function mechanism is known to improve the performance of TCP/IP networks by reducing queueing delays and desynchronizing flows. In this paper, we study yet another positive effect caused by this mechanism, i.e., the reduction in the clustering of packet losses, measured by the burst ratio. The main contribution consists of two new formulas for the burst ratio in systems with and without the dropping function, respectively. These formulas enable the easy calculation of the burst ratio for a general, non-Poisson traffic, and for an arbitrary form of the dropping function. Having the formulas, we provide several numerical examples that demonstrate their usability. In particular, we test the effect of the dropping function’s shape on the burst ratio. Several shapes of the dropping function proposed in the literature are compared in this context. We also demonstrate, how the optimal shape can be found in a parameter-depended class of functions. Finally, we investigate the impact of different system parameters on the burst ratio, including the load of the system and the variance of the service time. The most important conclusion drawn from these examples is that it is not only the dropping function that reduces the burst ratio by far; simultaneously, the more variable the traffic, the more beneficial the application of the dropping function.

## 1. Introduction

The basic queueing mechanism used commonly for packet queueing in Internet routers, i.e., the tail-drop FIFO queue, has several important drawbacks. First of all, it causes the infamous bufferbloat effect, meaning that there are long queues of packets in a significant fraction of buffers in the Internet. This not only adds substantial delays to packet delivery times but has also other negative consequences, such as the synchronization of TCP control loops among flows, unfair bandwidth allocation for flows with long RTT times, and others [1].

Recently, yet another disadvantage of simple tail-drop queueing was discovered and described. Namely, when the router’s buffer is full, several incoming packets can be dropped in a row, forming a sequence of losses [2,3]. Such sequences of losses are particularly bad for multimedia streams of real-time character, e.g., voice or video calls.

The solution of the bufferbloat problem, advocated strongly by the Internet Engineering Task Force, is the application of active queue management (AQM) in Internet routers [1]. In AQM, contrary to the tail-drop mechanism, an incoming packet can be dropped even if the router’s buffer is not full. Actually, it can be dropped even if the buffer occupancy is rather low. Moreover, the decision of whether an incoming packet should be accepted or dropped is probabilistic in nature, i.e., each packet can be accepted with some, dynamically changing, probability. Such randomization is supposed to mitigate the synchronization of TCP flows.

There are many active queue management algorithms proposed to date (see, e.g., [4,5,6,7,8] and the references given there). The main difference between them is how the dropping probability is computed based on current and past system parameters, such as the queue length and its dynamics, the packet loss events, the empty-system events, and others. In some AQM algorithms, advanced methods are exploited to compute the dropping probability, including neural networks [9,10], genetic algorithms [11], and fuzzy logic [12].

An important subclass of AQM algorithms is based on the dropping function. In such algorithms, the packet dropping probability is a function of the length of the queue of packets in the buffer. In fact, historically, the first AQM algorithm, RED [13], belongs to this class. In RED, a simple linear dropping function was used. After that, several other shapes of the dropping function have been tested, including the doubly linear shape [14], negative-exponential shape [15], quadratic shape [16], a mixture of third-degree polynomials and linear functions [17], and a product of logarithmic and linear functions [18].

The dropping function mechanism may not provide such excellent results as the mentioned methods based on machine learning techniques. It is, however, still worth our attention for at least two reasons. Firstly, it is very simple to implement in a networking device, contrary to advanced algorithms. Secondly, it still provides great improvements, if compared to the tail-drop mechanism.

There are no commercially available networking devices with the built-in dropping function mechanism. However, the mechanism has been recently implemented in an experimental device and tested in a real network [19]. Namely, the dropping function has been implemented in an ×86-multi-core server with DPDK cards and software, which enabled packet processing and forwarding speeds exceeding those required by the network itself. Then, the device was tested for over a month in the network of a large university, operating as usual. During this period, several thousands of performance measurements were gathered, at different times of the day and night and at different days within the week and weekend, i.e., under different traffic intensities and types. As shown in [19], various shapes of the dropping function provide significant shortening of queues and improvements in their stability while maintaining the packet loss rate at a similar level, as in the tail-drop case. Yet another observation made in [19] is a significant reduction in the burst ratio parameter by the dropping function. The burst ratio is a parameter that expresses how many times an average sequence of losses in the observed stream of packets is longer than an average sequence of losses in a hypothetical stream, in which all losses are uncorrelated. Therefore, the burst ratio characterizes the strength of the tendency of losses to form long sequences, which are particularly unwelcome in real-time multimedia transmissions.

It is intuitive and easy to understand why the burst ratio can be elevated in a network exploiting tail-drop queues in its nodes. Namely, several packets can be dropped in a row during continuous periods of time when the buffer is full (buffer overflow periods). This phenomenon was confirmed by direct measurements in a lab and in a real network, [2,3]. It was also explained theoretically, by the derivation of the burst ratio for tail-drop queueing models with various arrival stream types [20,21,22]. It is equally easy to understand why the dropping function mechanism can decrease the burst ratio. When this mechanism is used, the buffer overflow period occurs but is extremely rare or never occurs. In other words, there are no periods of time during which all arriving packets are lost. Instead, every packet can be randomly accepted or dropped, which makes it is much harder to form a sequence of consecutive losses.

Therefore, the state-of-the-art finding can be summarized as follows. It has been shown in networking experiments with a prototype device that the dropping function reduces the burst ratio significantly. Intuitively, this is expected. Thus far, however, there has been no deep mathematical analysis of this effect and no way to predict the extent of this effect precisely using numbers.

The purpose of this paper is to feel this gap, i.e., to make possible the calculation of the burst ratio in advance for a given parameterization of the traffic and a given form of the dropping function. The main contribution of this paper consists of explicite formulas for the burst ratio parameter in queueing models with the dropping function (Theorem 1) and without the dropping function (Theorem 2). What is important is that a general type of traffic, with the interarrival time distribution having an arbitrary form, is assumed. Having proven the formulas, we first study the impact of the dropping function on the burst ratio depending on the shape of the dropping function. Several shapes of the dropping function proposed in the literature are compared. We also demonstrate how the optimal shape can be found in a parameter-dependent class of functions. Then, we investigate the influence of other system parameters on the burst ratio, including the load of the system and the standard deviation of the service time.

To the author’s best knowledge, the results of this papers are new. Previously, the analysis of the burst ratio in a system with the dropping function was carried out only under the assumption that the traffic is Poisson in nature [23]. Such an assumption simplifies the analysis but renders the model less useful. Namely, it is well-known that the traffic in networks is far from Poisson and using Poisson approximation leads to optimistic underestimations of performance characteristics. Therefore, to obtain precise results, we have to to use the general distribution of the interarival time, as stated here. Other previous studies on the burst ratio, e.g., [20,21,22,24,25], do not incorporate the dropping function, which is the crucial component herein.

The remainder of the article is organized as follows. In Section 2, the formal description of the queueing model is given, as well as the definition and some properties of the burst ratio. In Section 3, two main theorems on the burst ratio are proven, and they are devoted to systems with and without the dropping function, respectively. In Section 4, numerical results are presented and discussed. They are divided into a few subsections, in which impacts of the shape of the dropping function, the load of the system, and the interarrival time distribution are studied. At the end of Section 4, results of simulations performed to verify the theoretical results are shown. Finally, some concluding remarks are provided in Section 5.

## 2. The Model

### 2.1. Queueing System

We deal with the single-server queueing model with the addition of the dropping function. Namely, packets arrive to the buffer, where they are stored before service (transmission) in the arrival order. The interarrival time distribution has distribution function G(t), which can have any form. We assume only that the average intararrival time is finite.

(1) $$EG=\int_{0}^{\infty }tdG\left(t\right)<\infty .$$

Systems with infinite interarrival times are not interesting; they have an average queue length of zero. The buffer size (system capacity) is finite and equals *K*, which means that the total number of packets that is present in the system cannot exceed *K*, including the service position. When the system is full upon a packet arrival, the newly arriving packet is dropped (deleted).

Moreover, the dropping function mechanism is applied, which means that an arriving packet can be dropped upon arrival even if the buffer is not full. This happens with probability d(n), where *n* is the queue length upon the arrival of the new packet, including the service position. Function d(n) is called the dropping function and can assume an arbitrary form, if only 0≤d(n)≤1 for every 0<n<K, d(0)<1 and d(n)=1 for n≥K. Assumption d(0)<1 excludes an uninteresting system with d(0)=1 in which every arriving packet is deleted, and the queue is always empty. Assumption d(n)=1 for n≥K is equivalent to the final-buffer assumption, as stated above.

The service time is exponentially distributed with parameter μ. Standard independence assumptions are made, i.e., all interarrval times and service times are mutually independent. The queue length at time *t* is denoted by X(t), which includes the service position, if occupied. We use the convention that the queue length process is left continuous, X(u−)=X(u). The load of the system is defined traditionally as follows.

(2) $$\rho =\frac{1}{\mu EG}.$$

It is easy to see that the presented model is equivalent to the G/M/1/K model in Kendall’s notation, but with the addition of the dropping function mechanism.

### 2.2. Clustering of Losses

The main characteristic studied in this paper is the burst ratio, which is introduced in [24].

Consider a stream of packets, some of them being deleted. The burst ratio, *B*, is the ratio of the average length of the sequence of packets deleted in a row in the considered stream to the average length of the sequence of losses in a hypothetical stream in which all deletions happen independently, with some probability *L*. Denoting the average length of the loss sequence in the observed stream by $\bar{G}$ while in the hypothetical stream it is denoted by $\bar{K}$, we have by definition the following.

(3) $$B=\frac{\bar{G}}{\bar{K}}.$$

It is a simple matter to compute $\bar{K}$ using the geometric series. We obtain $\bar{K}=1/\left(1-L\right)$. Therefore, (3) yields the following:

(4) $$B=\left(1-L\right)\bar{G},$$

where *L* is the overall packet loss probability.

In practice, we compute the burst ratio in two steps regardless of whether it is performed in experimental or theoretical studies. Firstly, we compute (or measure) the average length of the sequence of losses, $\bar{G}$. Secondly, we compute (or measure) the loss probability, *L*. Then, *B* is obtained from Formula (4).

Note that the two numbers, *L* and *B*, provide compact yet very informative characterization of the loss process. *L* informs us about the overall ratio of losses, while *B* informs us about their tendency to cluster together in sequences. If B=1, then there is no such tendency—the losses are random and uncorrelated. The greater *B* is above 1, the stronger the clustering of losses. In contemporary TCP/IP networks, *B* is typically significantly greater than 1 (see experimental studies of [2,3]). As it was explained in Section 1, this is caused by the tail-drop queueing mechanism.

Elevated values of both *L* and *B* have negative impact on real-time multimedia transmissions. In some cases, this effect can be measured precisely by using a strict formula. For instance, in [26], we can find a formula for the deterioration of the quality of IP voice calls as a function of *L* and *B* (see formula (7)–(29) of [26]).

## 3. Analysis

We will prove now two theorems on the burst ratio in systems with and without the dropping function, respectively.

**Theorem** **1.** *The burst ratio in the G/M/1/K system with dropping function d(n) is equal to the following:*

(5) $$B=\left(1-\sum_{n=0}^{K}p_{n}d\left(n\right)\right)\frac{\sum_{j=0}^{K}r_{j}{\bar{H}}_{j}}{\sum_{j=0}^{K}r_{j}},$$

*where*

(6) $$r_{0}=\sum_{i=0}^{K-1}p_{i}\left(1-d(i)\right)c_{i+1}d\left(0\right),$$

(7) $$r_{j}=\sum_{i=j-1}^{K-1}p_{i}\left(1-d(i)\right)b_{i+1-j}d\left(j\right),\;\;\;\;\;\;j\geq 1,$$

(8) $$b_{j}=\frac{1}{j!}\int_{0}^{\infty }e^{-\mu t}{\left(\mu t\right)}^{j}dG\left(t\right),\;\;\;\;\;c_{j}=1-\sum_{i=0}^{j-1}b_{i},$$

*${\bar{H}}_{n}$ is obtained recursively as follows:*

(9) $${\bar{H}}_{0}=\frac{1}{1-d(0)},$$

(10) $${\bar{H}}_{1}=\frac{1}{1-d\left(1\right)b_{0}}\left(1+\frac{d\left(0\right)c_{1}}{1-d(0)}\right),$$

(11) $${\bar{H}}_{n}=\frac{1}{1-d\left(n\right)b_{0}}\left(1+\sum_{m=1}^{n-1}d\left(m\right){\bar{H}}_{m}b_{n-m}+\frac{d\left(0\right)c_{n}}{1-d(0)}\right),\;\;\;\;\;\;n\geq 2,$$

*while*

(12) $$\left(p_{0},\ldots ,p_{K}\right)=\left(1,0,\ldots ,0\right)\cdot A^{-1},$$

*where matrix A has the form:*

(13) $$A={\left[a_{ij}\right]}_{i,j=0,\ldots ,K},\;\;\;a_{ij}=\left\{\begin{array}{cc} 1, & \text{if}\;\;0\leq i\leq K,j=0,\\ b_{i-j+1}\left(1-d\left(i\right)\right)+b_{i-j}d\left(i\right), & \text{if}\;\;0<i\leq K,0<j<i,\\ b_{1}\left(1-d\left(i\right)\right)+b_{0}d\left(i\right)-1, & \text{if}\;\;0<i\leq K,j=i,\\ b_{0}\left(1-d\left(i\right)\right), & \text{if}\;\;0\leq i\leq K-1,j=i+1,\\ 0, & \text{otherwise}. \end{array}\right.$$

**Proof.**  Proof of Theorem 1. Let $t_{1}$, $t_{2},\ldots$ denote consecutive arrival times and $X_{l}=X\left(t_{l}\right)$, l=1,2,…. Sequence $\left\{X_{l}\right\}$ constitutes a discrete-time Markov chain. This follows from the memorylessness property of the exponential distribution; namely, the remaining service time and the packet arrival are exponentially distributed with parameter μ no matter how much time the current service has already taken.

Take an arbitrary k>1 and assume that the queue length is *n* at time $t_{k}$. Let ${\bar{H}}_{n}$ denote the average number of consecutive losses from time $t_{k}$ under the condition that the packet arriving at time $t_{k}$ is lost. It must hold ${\bar{H}}_{n}\geq 1$, because the packet lost at time $t_{k}$ is included in ${\bar{H}}_{n}$. Moreover, due to the fact that $\left\{X_{l}\right\}$ is a Markov chain, the evolution of the system from time $t_{k}$ depends only on the queue length at time $t_{k}$ and does not depend on previous queue’s lengths. Therefore, ${\bar{H}}_{n}$ depends only on *n* and does not depend on *k*.

In the first part of the proof, we will prove Formulas (9)–(11) for ${\bar{H}}_{n}$. If n>0, then we have the following.

(14) $$\begin{array}{cc} {\bar{H}}_{n}=1+ & \sum_{m=1}^{n}d\left(m\right){\bar{H}}_{m}\int_{0}^{\infty }\frac{e^{-\mu t}{\left(\mu t\right)}^{n-m}}{(n-m)!}dG\left(t\right),\\ \; & +d\left(0\right){\bar{H}}_{0}\sum_{i=n}^{\infty }\int_{0}^{\infty }\frac{e^{-\mu t}{\left(\mu t\right)}^{i}}{i!}dG\left(t\right),\;\;\;\;\;\;\;\;\;0<n\leq K. \end{array}$$

Equation (14) can be explained as follows. Number 1 stands for the packet loss at time $t_{k}$. Let *m* be the queue length upon the next arrival time, $t_{k+1}$. If m≥1, then the probability of having the queue length *m* at time $t_{k+1}$ is given by the first integral in (14). This integral is obtained by conditioning on the duration of the interarrival time and using the Poisson formula for the probability of n−m completed services by time $t_{k+1}$. Then, the packet arriving at time $t_{k+1}$ can be lost with probability d(m) and the average number of consecutive losses from time $t_{k+1}$ is then ${\bar{H}}_{m}$. This explains the first row of (14). The second row corresponds to the case m=0. The probability of having queue length 0 at time $t_{k+1}$ is expressed now by the sum of integrals. Then, the packet arriving at time $t_{k+1}$ can be lost with probability d(0) and the average number of consecutive losses from time $t_{k+1}$ is ${\bar{H}}_{0}$.

If n=0, then we obtain the following:

(15) $${\bar{H}}_{0}=1+d\left(0\right){\bar{H}}_{0}.$$

Again, 1 stands for the packet loss at time $t_{k}$. The queue length just before the next arrival time must be 0; thus, the packet arriving at time $t_{k+1}$ is lost with probability d(0) and the average number of consecutive losses from time $t_{k+1}$ is ${\bar{H}}_{0}$ again.

From (15), we immediately obtain (9). Exploiting (9) and notation (8), Equation (14) can be rewritten as follows:

(16) $${\bar{H}}_{n}=1+\sum_{m=1}^{n}d\left(m\right){\bar{H}}_{m}b_{n-m}+\frac{d\left(0\right)c_{n}}{1-d(0)},\;\;\;\;\;\;\;\;\;0<n\leq K,$$

which for n=1, gives the following:

(17) $${\bar{H}}_{1}=1+d\left(1\right){\bar{H}}_{1}b_{0}+\frac{d\left(0\right)c_{1}}{1-d(0)},$$

while for n≥2, the following is obtained.

(18) $${\bar{H}}_{n}=1+d\left(n\right){\bar{H}}_{n}b_{0}+\sum_{m=1}^{n-1}d\left(m\right){\bar{H}}_{m}b_{n-m}+\frac{d\left(0\right)c_{n}}{1-d(0)}.$$

Finally, from (17) and (18), we obtain (10) and (11), respectively.

In the second part of the proof, we will derive the overall loss probability, *L*.

We start with computing the transition matrix for the Markov chain $\left\{X_{l}\right\}$. Firstly, the probability of the transition from non-zero state *i* to non-zero state j≤i is equal to the following. $\left(1-d\left(i\right)\right)b_{i-j+1}+d\left(i\right)b_{i-j}.$

Indeed, such a transition can happen in two ways—either the first packet is accepted and i−j+1 services are completed by the next arrival time, or the first packet is dropped and i−j services are completed by the next arrival time. Secondly, the probability of the transition from state i<K to state i+1 equals the following. $\left(1-d\left(i\right)\right)b_{0}.$

Indeed, the first packet must be accepted and no service can be completed by the next arrival time in this case. Finally, the probability of the transition from any state to state 0 is as follows.

$$\left(1-d\left(i\right)\right)\left(1-\sum_{j=0}^{i}b_{j}\right)+d\left(i\right)\left(1-\sum_{j=0}^{i-1}b_{j}\right).$$

Other transitions are not possible. Summarizing these considerations, we obtain the transition matrix *Q* in the following form:

(19) $$Q={\left[q_{ij}\right]}_{i,j=0,\ldots ,K},\;\;\;q_{ij}=\left\{\begin{array}{cc} c_{i+1}\left(1-d\left(i\right)\right)+c_{i}d\left(i\right) & \text{if}\;\;0\leq i\leq K,j=0,\\ b_{i-j+1}\left(1-d\left(i\right)\right)+b_{i-j}d\left(i\right), & \text{if}\;\;0<i\leq K,0<j\leq i,\\ b_{0}\left(1-d\left(i\right)\right), & \text{if}\;\;0\leq i\leq K-1,j=i+1,\\ 0, & \text{otherwise}. \end{array}\right.$$

The stationary vector $p=(p_{0},\ldots ,p_{K})$ for this chain can be obtained in the standard method by solving the system of equations:

(20) $$\left\{\begin{array}{c} pQ=p,\\ \sum_{j=0}^{K}p_{j}=1. \end{array}\right.$$

Matrix *Q* is known to be linearly dependent; thus, the Equation in (20) corresponding to the first column of *Q* can be removed. Then, rearranging the remaining equations and grouping $\left(p_{0},\ldots ,p_{K}\right)$ on the left side, we obtain an explicit solution of (20) in (12) and (13).

Now, due to the fact that $p_{n}$ is the stationary probability that the queue length upon a packet arrival is *n*, we can compute the loss probability as follows.

(21) $$L=\sum_{n=0}^{K}p_{n}d\left(n\right).$$

In the third part of the proof, we will derive the average length of the sequence of consecutive losses, $\bar{G}$, using ${\bar{H}}_{n}$. Note first that it is not quite trivial to obtain $\bar{G}$ from ${\bar{H}}_{n}$, because in the definition of ${\bar{H}}_{n}$, it is not assumed that the sequence of losses begins at time $t_{k}$—it may begin before $t_{k}$.

To overcome this, consider a sequence of losses that begins at arrival time $t_{k}$, when the system is in the stationary regime and $X_{k}=j$. Such a sequence can be initiated only if the previous packet was accepted to the buffer. In particular, the previous packet must have arrived to the buffer at time $t_{k-1}$, when the queue length was *i* and j−1≤i<K, it must have been accepted with probability 1−d(i), and the queue length must have changed from *i* to *j* during the interarrival time. It is easy to see that for j>0, such a series of events happens with the following probability:

(22) $$p_{i}\left(1-d(i)\right)b_{i+1-j}d\left(j\right),$$

while for j=0, such a series of events happens with the following probability.

(23) $$p_{i}\left(1-d(i)\right)c_{i+1}d\left(0\right).$$

Now, define $r_{j}$ as the probability that, at an arbitrary arrival time, the queue length is *j* and a sequence of losses begins at that time. From the considerations of the previous paragraph and (22) and (23), we obtain Formulas (7) and (6) for $r_{j}$, respectively. The probability that a sequence of losses begins at arbitrary arrival time is then $\sum_{j=0}^{K}r_{j}$. Therefore, we can conclude that the average length of a sequence of losses, which begins at arbitrary arrival time, is as follows.

(24) $$\bar{G}=\frac{\sum_{j=0}^{K}r_{j}{\bar{H}}_{j}}{\sum_{j=0}^{K}r_{j}}.$$

Finally, combining (4) with (21) and (24), we arrive at (5), which completes the proof. □

Now we can prove an analog of Theorem 1 but for the system without the dropping function.

**Theorem** **2.** *The burst ratio in the G/M/1/K system is equal to the following:*

(25) $$B=\frac{1-p_{K}}{1-b_{0}},$$

*where $b_{j}$ is given in (8) while the following is the case:*

(26) $$\left(p_{0},\ldots ,p_{K}\right)=\left(1,0,\ldots ,0\right)\cdot A^{-1},$$

*and matrix A is provided as follows:*

(27) $$A={\left[a_{ij}\right]}_{i,j=0,\ldots ,K},\;\;\;a_{ij}=\left\{\begin{array}{cc} 1, & \text{if}\;\;0\leq i\leq K,j=0,\\ b_{i-j+1}, & \text{if}\;\;0<i<K,0<j<i,\\ b_{K-j}, & \text{if}\;\;i=K,0<j<K,\\ b_{1}-1, & \text{if}\;\;0<i<K,j=i,\\ b_{0}-1, & \text{if}\;\;i=j=K,\\ b_{0}, & \text{if}\;\;0\leq i\leq K-1,j=i+1,\\ 0, & \mathrm{otherwise}. \end{array}\right.$$

**Proof.**  Proof of Theorem 2. This theorem can be proven at least in two different ways.

In one proof, we can use Theorem 1 and apply a trivial dropping function, i.e., d(n)=0 for n<K and d(n)=1 otherwise. Obviously, such a dropping function renders the system equivalent to the system without the dropping function, but with a finite buffer. Using this trivial dropping function, we obtain the following from (6) and (7):

(28) $$r_{j}=0,\;\;\;0\leq j<K,$$

while from (9) to (11), the following is obtained

(29) $${\bar{H}}_{n}=1,\;\;\;0\leq n<K,$$

(30) $${\bar{H}}_{K}=\frac{1}{1-b_{0}},$$

and from (21), we have the following.

(31) $$L=p_{K}.$$

Formulas (28)–(31) combined with (5) lead to (25), where matrix *A* in (27) is just a simplified matrix (13).

Alternatively, the proof can be conducted without references to Theorem 1 using probabilistic arguments. We have to notice two facts. Firstly, a sequence of losses in the system can occur only during the buffer’s overflow period. Secondly, the duration of the buffer’s overflow period is exponentially distributed with parameter μ. This is a consequence of the memorylessness property of the exponential distribution, i.e., no matter when the buffer is overflowed, the remaining service time is exponentially distributed. On the other hand, the buffer overflow period is equal to the remaining service time upon the buffer’s overflow. Now, consider the beginning of the overflow period, i.e., the arrival time, when the queue length jumps from K−1 to *K*. With probability $b_{0}$, the next arrival will happen before the end of the overflow period. In this case, the new packet is lost and the sequence of losses is extended by 1. What is more, the probability of having yet another arrival before the end of the overflow period is again $b_{0}$ due to the memorylessness property of the exponential distribution. Therefore, we have in fact a series of Bernoulli experiments, in which the probability of a failure (loss) in a single experiment is $b_{0}$. Therefore, the average length of a sequence of failures (losses) is equal to $\frac{1}{1-b_{0}}$. This, combined with the obvious relation $L=p_{K}$ and (4), gives (25), while matrix *A* can be easily obtained by using transition probabilities of chain $\left\{X_{l}\right\}$. □

## 4. Examples

In the numerical examples, we use the following forms of the dropping function.

$$d_{1}\left(n\right)=\left\{\begin{array}{ccc} 0, & \text{if} & n<20,\\ 0.005n-0.1, & \text{if} & 20\leq n<40,\\ 0.09n-3.5, & \text{if} & 40\leq n<50,\\ 1, & \text{if} & n\geq 50, \end{array}\right.$$

$$d_{2}\left(n\right)=\left\{\begin{array}{ccc} 0, & \text{if} & n<20,\\ \frac{{\left(n-20\right)}^{2}}{900}, & \text{if} & 20\leq n<50,\\ 1, & \text{if} & n\geq 50, \end{array}\right.$$

$$d_{3}\left(n\right)=\left\{\begin{array}{ccc} 0, & \text{if} & n<20,\\ \frac{n-20}{30}, & \text{if} & 20\leq n<50,\\ 1, & \text{if} & n\geq 50, \end{array}\right.$$

$$d_{4}\left(n\right)=\left\{\begin{array}{ccc} 0, & \text{if} & n<20,\\ \frac{{\left(n-20\right)}^{3}}{3000}, & \text{if} & 20\leq n<30,\\ \frac{n-20}{30}, & \text{if} & 30\leq n<40,\\ \frac{{\left(n-50\right)}^{3}}{3000}+1, & \text{if} & 40\leq n<50,\\ 1, & \text{if} & n\geq 50, \end{array}\right.$$

$$d_{5}\left(n\right)=\left\{\begin{array}{ccc} 0, & \text{if} & n<20,\\ 1.055(1-e^{-(n-20)/10}), & \text{if} & 20\leq n<50.\\ 1, & \text{if} & n\geq 50. \end{array}\right.$$

**Figure 1:**
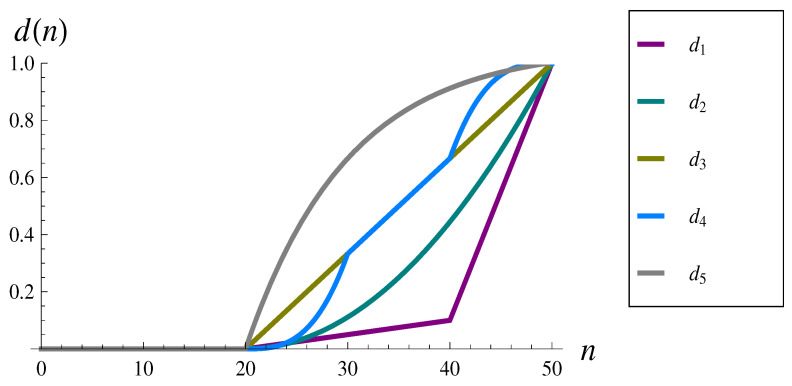
Dropping functions $d_{1}$–$d_{5}$.

They are also depicted in Figure 1. As observed, the doubly linear, quadratic, linear, combined cubic, and negative exponential shapes of the dropping function are used. Such shapes of the dropping function are not new. They were proposed in the literature by the authors of [13,14,15,16,17]. In none of these papers, however, was there a study of the impact of the dropping function on the burst ratio carried out. The particular parameterizations are based on the assumption that the dropping function operates roughly at the second half of the buffer size. Assuming a buffer size of 50 packets, the dropping function operates herein on interval [20,50]. In every case, the dropping begins with zero probability at point (20,0), and ends with the probability of 1 at point (50,1). This enables obtaining sample parameterizations of the dropping function as presented above.

As it was already mentioned, in every case with the dropping function, the buffer size is K=50. The same buffer size is used in examples with no dropping function. If not stated otherwise, the interarrvial time distribution is gamma with parameters α=β=0.25, i.e., with the mean of 1 and the standard deviation of 2. This is altered in Section 4.3, where the impact of the interarrival time on the burst ratio is studied and in simulations shown in Section 4.4. Similarly, if not stated otherwise, the service rate is μ=1, resulting in ρ=1. This is altered in Section 4.2, where the influence of the system load is investigated and in simulations in Section 4.4.

### 4.1. Impact of d(n) Shape

In this subsection, we will check the influence of the shape and aggressiveness of the dropping function on the burst ratio.

In Table 1, burst ratios $B_{1}$–$B_{5}$ computed for functions $d_{1}\left(n\right)$–$d_{5}\left(n\right)$ are presented, as well as the burst ratio computed without the dropping function, $B_{0}$. Additionally, every $B_{i}$ is presented as a percentage of $B_{0}$. The numbers were obtained using Theorems 1 and 2.

The following conclusions can be drawn from Table 1. Firstly, a high burst ratio is observed in the case with no dropping function. Secondly, the application of the dropping function reduces its value significantly. Namely, $B_{0}$ is reduced to 44–50% of its original value, when the dropping function is used. There are some differences among $d_{1}$–$d_{5}$, but all of them provide a great improvement.

To analyze in detail the impact of particular shape of the dropping function, we note first that $d_{1}$–$d_{5}$ are numbered roughly from the less aggressive, $d_{1}$, to the most aggressive, $d_{5}$ (see Figure 1). The best burst ratio is obtained for a rather mild function $d_{2}$. The general picture is, however, rather complicated. Function $d_{1}$ performs worse than $d_{2}$, even though $d_{1}$ is less aggressive in general. One can argue that this is because $d_{1}$ is more aggressive in the initial interval [20,23]. On the other hand, $d_{4}$ is even less aggressive than $d_{2}$ in this interval, but it provides worse *B*. It is also interesting to compare the results computed for $d_{3}$ and $d_{4}$, because the latter is less aggressive in the interval [20,30] than the former, the same in the interval [30,40], and more aggressive in the interval [40,50]. As we see in Table 1, $d_{3}$ performs better than $d_{4}$.

**Figure 2:**
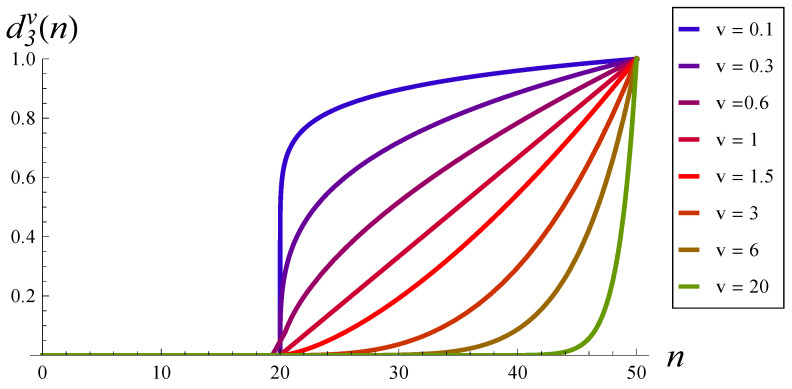
Dropping functions $d_{3}^{v}\left(n\right)$ for *v* from 0.1 to 20.

To study the impact of the dropping function shape more systematically, we can use a class of functions $d_{3}^{v}$, where exponent *v* is a parameter. A few selected shapes of function $d_{3}^{v}$, for *v* from 0.1 to 20, are presented in Figure 2.

**Figure 3:**
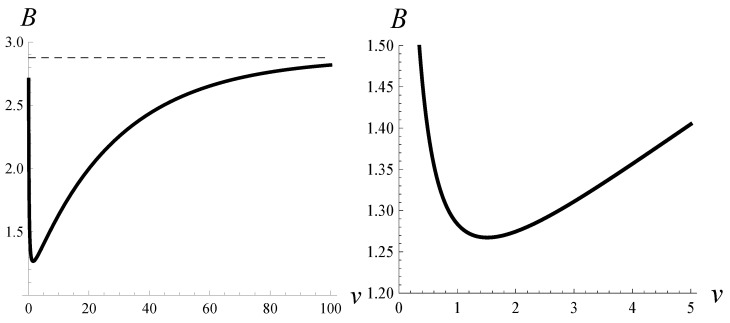
Burst ratio versus parameter *v* when dropping function $d_{3}^{v}\left(n\right)$ is applied.

In Figure 3 and Table 2, the burst ratio as a function of parameter *v* is shown.

It is easy to observe that if v→∞, then function $d_{3}^{v}\left(n\right)$ converges to the unit-step function with the step at n=50. Therefore, we can see in Figure 3 that the burst ratio approaches a limit when v→∞. The limiting value is 2.8764 (see Table 2), and it is equal to the burst ratio in a system without the dropping function and the buffer of size 50. Similarly, if v→0+, then function $d_{3}^{v}\left(n\right)$ converges to the unit-step function with the step at n=20. Therefore, if v→0+, then the burst approaches the value for a system without the dropping function and the buffer of size 20. This value is 2.6906; see Table 2.

What is important is that we can observe a minimum for v=1.5 in Figure 3. The optimal value of the burst ratio, achieved for v=1.5, is equal to 1.2674 (see Table 2). The shape of the dropping function that provides the minimal value of the burst ratio is depicted in Figure 2 in red.

Now, it is hard to explain why this particular dropping function in class $d_{3}^{v}$ provides the best burst ratio. In Theorem 1, we see that the burst ratio depends in a very complicated way on the dropping function. The form of this function influences the stationary distribution of the queue length, the loss probability and the structure of losses, each of them having impact on each other and on the resulting burst ratio. Fortunately, we can always use Theorem 1 to find the optimal shape of the dropping function with respect to the burst ratio in a parameter-dependent class of functions, as shown above.

### 4.2. Impact of ρ

In the next set of numerical results, we study the influence of the system load on the burst ratio. By varying the service rate, μ, we change the load of the system from a severely underloaded one (ρ=0.5) to a severely overloaded one (ρ=2.0) and check how it influences the burst ratio when dropping functions $d_{1}$–$d_{5}$ are applied, as well as when there is no dropping function.

**Figure 4:**
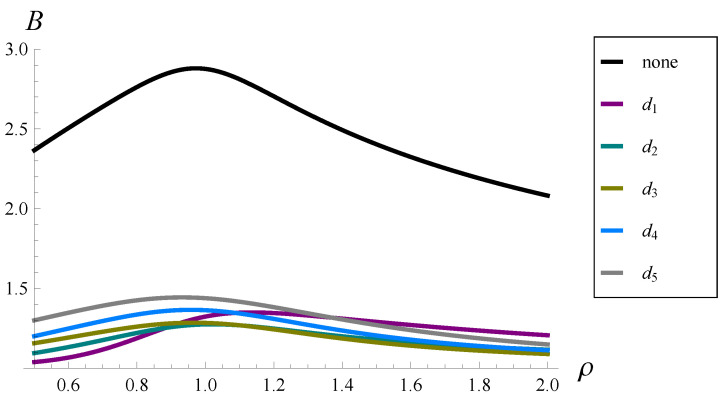
Burst ratio versus the system load for dropping functions $d_{1}$–$d_{5}$ and no dropping function.

The results are presented in Figure 4 and Table 3. As we can notice, a high value of the burst ratio is achieved in a system with no dropping function, in the entire interval of the load, from 0.5 to 2.0. Moreover, there is a maximum on the black curve in Figure 4 close to ρ=1. When any form of the dropping function is applied, the burst ratio is reduced hlsignificantly, again in the entire interval, which is clearly seen in both Figure 4 and Table 3.

In Table 4, $B_{i}$ as a percentage of $B_{0}$ is presented. As we can see, the application of the dropping reduces the burst ratio to 43–58% of its original value, depending on the dropping function form and the system load.

Two more interesting observations can be made.

Firstly, the curves for $d_{1}$–$d_{5}$ in Figure 4 intersect a few times. This means that it depends on the system load, and when shaped among $d_{1}$–$d_{5}$, it performs better; otherwise, it performs worse. For ρ=0.5, in particular, $d_{1}$ is the best, followed by $d_{2}$, $d_{3}$, $d_{4}$, and $d_{5}$. For ρ=1, $d_{2}$ is the best, followed by $d_{3}$, $d_{1}$, $d_{4}$, and $d_{5}$. For ρ=2, $d_{3}$ is the best, followed by $d_{4}$, $d_{2}$, $d_{5}$, and $d_{1}$. Therefore, we cannot say that one form of the dropping function is better than the other in general—their performance may vary with ρ, which may change in TCP/IP networks. Fortunately, all functions $d_{1}$–$d_{5}$ provide similarly low values of *B* when compared to the system with no dropping function in the entire range of ρ. Hence, which particular shape is applied is not that important. It is important to apply one of them.

Second, if the dropping function is applied, then the local maximum of the burst ratio can be assumed for ρ and not necessarily close to 1. For instance, we see in Figure 4 that in the case of $d_{5}$, the maximum is achieved for ρ<1, while in the case of $d_{1}$, for ρ>1. Similarly, in Table 3 we see that the burst ratio for ρ=1.25 is greater than for ρ=1.

Finding analytically the maximum of the burst ratio as a function of ρ seems to be very hard. Fortunately, this maximum does not have a great practical meaning, because all curves for dropping functions $d_{1}$–$d_{5}$ are rather flat. Moreover, in practice, we do not have such large loads, such as 2. Thus, the interval of interest is shorter, making the curves even flatter. Finally, there is no need to analyze the burst ratio for a very small load, e.g., 0.1. In such cases, losses are very rare, so the value of the burst ratio is practically insignificant.

### 4.3. Impact of DG

In this set of numerical results, we study the impact of the interarrival time distribution on the burst ratio. We focus on the value of the standard deviation of the interarrival time, DG (varying the average interarrival time, EG, changes the system load, for which its influence was already studied).

Therefore, we use the gamma distribution of the interarrival time, with α=β=w, where w>0 is a parameter. Hence, EG=1 for every *w*, while DG is a function of *w*, namely $DG=w^{-1/2}$.

**Figure 5:**
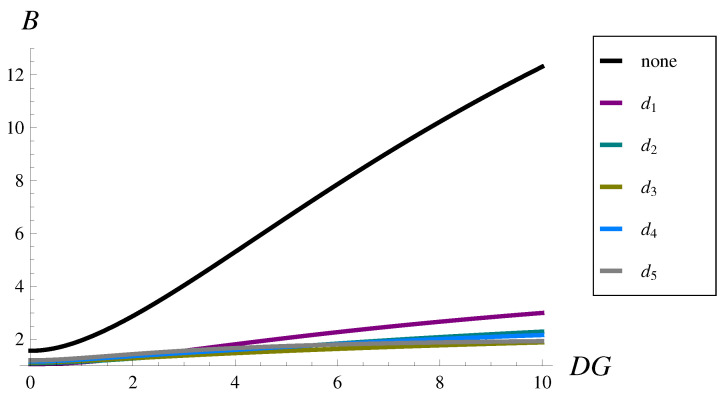
Burst ratio versus the standard deviation of the interarrival time for dropping functions $d_{1}$–$d_{5}$ and no dropping function.

The results are presented in Figure 5 and Table 5. They were obtained by varying *w* in such a way that the resulting DG varied from 0 to 10.

As we can see in Figure 5, the burst ratio depends strongly on the standard deviation of the interarrival time. In the case with no dropping function and highly variable traffic, large values of the burst ratio are obtained above 12.3 (see Table 5). This means that the average sequence of losses is 12.3-times longer than in the case of uncorrelated losses.

**Figure 6:**
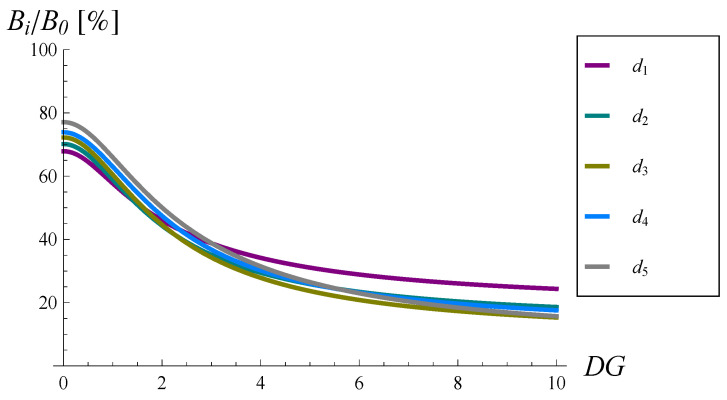
$B_{i}$ as a percentage of $B_{0}$ versus the standard deviation of the interarrival time.

Fortunately, every considered dropping function decreases the burst ratio significantly. Moreover, the larger the DG, the greater the relative improvement of the burst ratio. This is demonstrated in Figure 6, in which $B_{i}$ as a percentage of $B_{0}$ is depicted. For instance, if DG=2, then the burst ratio is reduced to 43–58% of its original value depending on the dropping function form. For DG=4, the burst ratio is reduced to 24–28% of its original value, while for DG=10, the burst ratio is reduced to 15–24% of its original value, depending on the dropping function form.

### 4.4. Verification via Simulations

To make sure that the theoretical results of Theorems 1 and 2 are error free, they were also verified using simulations. For this purpose, Omnet++ discrete-event simulator was exploited [27]. Tens of simulation runs were carried out, with different forms of the dropping function, interarrival time distributions and system loads. In every simulation run, 100 million simulated packets passed through the system. In every case, a high agreement between simulated and theoretical result was obtained.

In Table 6, a few of these results are presented, obtained for dropping functions $d_{1}$–$d_{5}$ and no dropping function; the interarrival time is parameterized as in Section 4.3, and the load varied from 0.9 to 1.4. As we can see, the simulated and theoretical values are very close to each other. A slightly worse result for ρ=0.9 can be associated with the fact that there were relatively few losses observed in this simulation run for an obvious reason. Nonetheless, the resulting relative error is still less than $1/10^{4}$.

## 5. Conclusions

In this paper, an analysis of the burst ratio parameter in a queueing system with the dropping function was carried out.

The study was motivated by two facts. Firstly, the dropping function is one of the important tools of choice in active queue management, which is recommended by IETF to reduce queueing delays and to desynchronize TCP control loops in the Internet. Secondly, it has been observed in recent experiments that the application of the dropping function reduces the burst ratio as well, which is a nice side effect. The burst ratio reflects the tendency of packet losses to form long sequences, which are especially bad for real-time multimedia transmissions.

The main contribution of this paper consists of two new theorems on the value of the burst ratio. They enable an easy calculation of the burst ratio value in systems with an arbitrary distribution of the interarrival time, an arbitrary form of the dropping function, and with no dropping function. They enable also finding the optimal shape of the dropping function, in terms of the minimal burst ratio, in a parameter-dependent class of functions.

Theoretical results were illustrated with numerical examples based on several dropping function types proposed in the literature. In particular, the influence of the dropping function shape, the system load, and the standard deviation of the service time on the burst ratio was investigated.

At least three important observations were made.

First, the application of the dropping function reduced the burst ratio significantly in all considered scenarios. In most scenarios, with moderate variability of the interarrival time, the reduced burst burst ratio was about 50% smaller than the original burst ratio obtained without the dropping function (see Table 1 and Table 4).

Second, the worse was the traffic in terms of the variability of the interarrival time: the more variable the interarrival time, the more beneficial the application of the dropping function, i.e., the greater the reduction. For instance, when the coefficient of variation was 10, the burst ratio reduced to 15–24% of its original value (see Figure 6).

Third, the differences in the performance offered by different shapes of the dropping function, proposed in the literature, were not significant—all of them provided a similar, high reduction of the burst ratio when compared to no dropping function.

## Figures and Tables

**Table 1:** Burst ratio $B_{i}$ for *i*-th dropping function in the first row. In the second row, $B_{i}$ is a percentage of $B_{0}$.

Dropping Function	None	$d_{1}\left(n\right)$	$d_{2}\left(n\right)$	$d_{3}\left(n\right)$	$d_{4}\left(n\right)$	$d_{5}\left(n\right)$
$B_{i}$	2.8764	1.3236	1.2746	1.2839	1.3634	1.4389
$B_{i}/B_{0}$	100%	46%	44%	45%	47%	50%

**Table 2:** Burst ratio for selected values of parameter *v*.

*v*	0	0.1	0.5	1.5	5	10	50	100	*∞*
*B*	2.6906	2.015	1.3953	1.2674	1.4047	1.6360	2.5622	2.8186	2.8764

**Table 3:** Burst ratio for selected values of the system’s load.

	ρ=0.5	ρ=0.75	ρ=1	ρ=1.25	ρ=1.5	ρ=1.75	ρ=2
no drop. fun.	2.3660	2.7029	2.8764	2.6484	2.4036	2.2212	2.0819
$d_{1}\left(n\right)$	1.0383	1.1499	1.3236	1.3390	1.2904	1.2442	1.2064
$d_{2}\left(n\right)$	1.0947	1.1989	1.2746	1.2361	1.1794	1.1412	1.1160
$d_{3}\left(n\right)$	1.1566	1.2455	1.2839	1.2287	1.1616	1.1167	1.0888
$d_{4}\left(n\right)$	1.2006	1.3174	1.3634	1.2903	1.2049	1.1477	1.1122
$d_{5}\left(n\right)$	1.2999	1.4104	1.4389	1.3633	1.2700	1.1987	1.1488

**Table 4:** Burst ratio $B_{i}$ as a percentage of $B_{0}$ for different values of the system load.

	ρ=0.5	ρ=0.75	ρ=1	ρ=1.25	ρ=1.5	ρ=1.75	ρ=2
$B_{1}/B_{0}$	44%	43%	46%	51%	54%	56%	58%
$B_{2}/B_{0}$	46%	44%	44%	47%	49%	51%	54%
$B_{3}/B_{0}$	49%	46%	45%	46%	48%	50%	52%
$B_{4}/B_{0}$	51%	49%	47%	49%	50%	52%	53%
$B_{5}/B_{0}$	55%	52%	50%	51%	53%	54%	55%

**Table 5:** Burst ratio for selected values of the standard deviation of the interarrival time.

	DG=0	DG=2	DG=4	DG=6	DG=8	DG=10
no drop. fun.	1.5662	2.8764	5.3032	7.8608	10.2240	12.3063
$d_{1}\left(n\right)$	1.0629	1.3236	1.8112	2.2706	2.6652	2.9969
$d_{2}\left(n\right)$	1.0987	1.2746	1.5621	1.8400	2.0836	2.2907
$d_{3}\left(n\right)$	1.1313	1.2839	1.4761	1.6377	1.7716	1.8832
$d_{4}\left(n\right)$	1.1569	1.3634	1.6010	1.8218	2.0098	2.1609
$d_{5}\left(n\right)$	1.2071	1.4389	1.6685	1.8043	1.8837	1.9317

**Table 6:** Simulated versus theoretical burst ratios for different dropping functions, loads, and interarrival time standard deviations.

System Parameters	Simulation	Theory
no drop. f., ρ=0.9, DG=4.0	5.3724	5.3719
$d_{1}\left(n\right)$, ρ=1.0, DG=3.0	1.5649	1.5647
$d_{2}\left(n\right)$, ρ=1.1, DG=2.0	1.2689	1.2688
$d_{3}\left(n\right)$, ρ=1.2, DG=1.0	1.1316	1.1316
$d_{4}\left(n\right)$, ρ=1.3, DG=0.5	1.0972	1.0972
$d_{5}\left(n\right)$, ρ=1.4, DG=0.25	1.0924	1.0924

## Data Availability

Not applicable.
